# Supplementary material for: Impact of parents’ physical activity on preschool children’s physical activity: a cross-sectional study
Source: PeerJ. 2018 Feb 27;6:e4405. doi: 10.7717/peerj.4405 (PMC5833469; doi:10.7717/peerj.4405)
Supplement: Supplemental Information 1 [file peerj-06-4405-s002.doc]

**填表日期 年 月 日**

**上海市学龄前儿童家长健康行为调查问卷**

尊敬的家长：

您现在参与的是《上海市学龄前儿童生活方式对体质健康及认知发展影响》的调查，该问卷将了解父母双方的基本信息，身体活动及健康行为以及子女相关情况。本次在上海地区的调查将有450名左右的学龄前儿童及其家庭参与。您的回答将会增加对上海市学龄前儿童身体活动和生活方式的了解。

所有的信息都会进行严格的**保密**。回答问卷是完全**自愿**的，并且您的回答也将会被**保密**。请您仔细阅读题目并如实回答。除个别注明为多选题外，其余均为单选题。请按顺序**答完所有题目**。

非常感谢您的参与和合作！

XXXXX - XXXXX幼稚园

孩童体质健康科研基地

XXXX年XX月

儿童姓名：   性别： 班级： 您的联系方式：

**以下部分请由父亲填写**

**1. 家长加速度仪器佩戴者:**

1□ 父亲 2□ 母亲

3□ 其他监护人   （如爷爷、奶奶、外公、外婆等，请填写）

**2. 您的户籍类型是？**

1□上海市城镇户口 2□上海市农业户口 3□其他省市农业户口 4□港澳台及境外

**3. 您的最高学历是？**

1□ 初中及以下 2□ 高中或中专 3□ 大专　 4□ 本科

5□ 硕士研究生 6□ 博士研究生

**4. 您主要的工作场所？**

1□ 办公室　 2□ 厂矿车间　 3□ 田间（包括草、牧、林、渔场）

4□ 柜台、摊位、餐厅　 5□ 交通运输环境（飞机、火车、轮船、公交等）

6□ 广场、街道、公路、铁路　 7□ 其它

1. **您目前的月收入是？**

1□ 无收入 2□ 2000元以下 3□ 2000-4000元

4□ 4001-8000元 5□ 8001-15000 元 6□ 15000元及以上

1. **您居住小区的名称是 ，**

**您入住该小区的年限大约有 年 （不到1年填0.5）**

**7. 您现在居住的小区属于那种类型？**

1□ 传统街坊社区（里弄住宅、花园住宅、老式公寓）

2□ 单位公房社区（同一单位居住区、新村、售后公房）

3□ 一般商品房社区 4□ 高档商品房社区 5□ 农村住房

**8．您住在 层，您所住的楼共 层**

**9．您上下楼的方式是**

1□ 坐电梯 2□ 走楼梯 3□ 混合(请详细说明)

**以下是关于您身体活动情况的调查，请先仔细看完不同类型活动的说明再填写。**


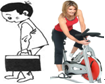
**说明：**

**剧烈活动**，即强而有力的身体活动，是指以费力的身体负荷且让您呼吸较正常更为急促的活动，仅回想您所做过**每次至少10分钟**的那些身体活动，如提重物、苦力、有氧运动或快骑脚踏车等；

**
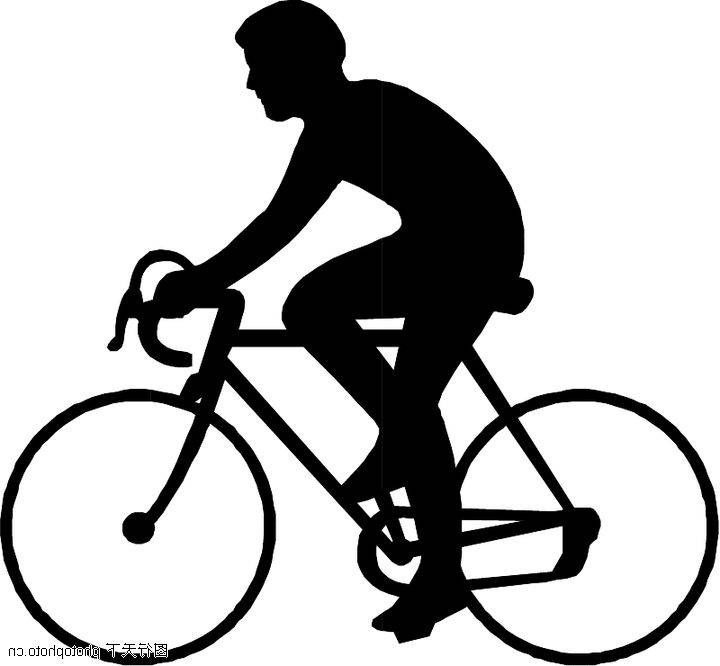
**

**中等强度活动**是指以适度的身体负荷让您呼吸比正常费力一些的活动，如提轻的物品、正常的速度骑脚踏车或网球双打等，但**不包含走路**；

**
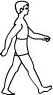
**

**走路**包含工作、在家、从某地到某地、娱乐、游戏或休闲时的走路。

**10. 在最近的7天里，您有几天进行以下活动？这7天里，平均每天每项活动的时间分别为？**

|  | 0天 | 1天 | 2天 | 3天 | 4天 | 5天 | 6天 | 7天 | 平均每天活动时间为 |
| --- | --- | --- | --- | --- | --- | --- | --- | --- | --- |
| 剧烈活动 | □ | □ | □ | □ | □ | □ | □ | □ | 小时 分钟 |
| 中等强度活动 | □ | □ | □ | □ | □ | □ | □ | □ | 小时 分钟 |
| 至少走10分钟的路 | □ | □ | □ | □ | □ | □ | □ | □ | 小时 分钟 |

**11. 工作期间（如朝九晚五，8小时），您通常每天静坐的时间约为 小时 分钟。**

**12. 在非工作时间里，您通常每天静坐（主要包括看电视、使用电脑和手机）的时间为？请针对工作日间和周末分别回答**

|  | | 时 间 | 总 计 |
| --- | --- | --- | --- |
| **工**  **作**  **日** | 看电视、电影、阅读 | 约 小时 分钟 | 约 小时 分钟 |
|  | 玩电脑、手机  (浏览网页、聊天、游戏、办公等) | 约 小时 分钟 |  |
|  | 其它（请详写） | 约 小时 分钟 |  |
| **周**  **末** | 看电视、电影、阅读 | 约 小时 分钟 | 约 小时 分钟 |
|  | 玩电脑、手机  (浏览网页、聊天、游戏、办公等) | 约 小时 分钟 |  |
|  | 其它（请详写） | 约 小时 分钟 |  |

**13. 您通常每天睡眠（晚上和午睡）多长时间？ 小时 分钟**

**14. 您在过去的半年内……？ *请在每栏里选择一个答案***

|  | 1） | 2） | 3） |
| --- | --- | --- | --- |
|  | 没有 | 偶尔 | 时常 |
| 说梦话或者梦游吗 | □ | □ | □ |
| 做过噩梦吗？ | □ | □ | □ |
| 入睡困难吗？ | □ | □ | □ |
| 后半夜起来后,再次入睡困难吗？ | □ | □ | □ |
| 入睡容易且能睡整晚并中间不醒来吗？ | □ | □ | □ |
| 经常感觉到疲劳 | □ | □ | □ |
| 行动迟缓，缺乏活力 | □ | □ | □ |

**15. 您与孩子接触的方式主要为**

1□ 谈话 2□ 游戏 3□ 辅导作业 4□ 打电话 5□ 任其自由

6□ 其他

**16. 您与孩子每天接触的时间为？** 请针对工作日和周末分别回答

**周末**

1□ 没有

2□ 大约每天半小时

3□ 大约每天一小时

4□ 大约每天两小时

5□ 大约每天三小时

6□ 大约每天四小时

7□ 大约每天五小时

8□ 大约每天六小时

9□ 大约每天七小时或是更多

**工作日**

1□ 没有

2□ 大约每天半小时

3□ 大约每天一小时

4□ 大约每天两小时

5□ 大约每天三小时

6□ 大约每天四小时

7□ 大约每天五小时

8□ 大约每天六小时

9□ 大约每天七小时或是更多

**以下部分请由母亲填写**

**1. 您的户籍类型是？**

1□上海市城镇户口 2□上海市农业户口 3□其他省市农业户口 4□港澳台及境外

**2. 您的最高学历是？**

1□ 初中及以下 2□ 高中或中专 3□ 大专　 4□ 本科

5□ 硕士研究生 6□ 博士研究生

**3. 您主要的工作场所？**

1□ 办公室　 2□ 厂矿车间　 3□ 田间（包括草、牧、林、渔场）

4□ 柜台、摊位、餐厅　 5□ 交通运输环境（飞机、火车、轮船、公交等）

6□ 广场、街道、公路、铁路　 7□ 其它

**4. 您目前的月收入是？**

1□ 无收入 2□ 2000元以下 3□ 2000-4000元

4□ 4001-8000元 5□ 8001-15000 元 6□ 15000元及以

**5．您上下楼的方式是**

1□ 坐电梯 2□ 走楼梯 3□ 混合(请详细说明)

**以下是关于您身体活动情况的调查，请先仔细看完不同类型活动的说明再填写。**


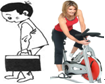
**说明：**

**剧烈活动**，即强而有力的身体活动，是指以费力的身体负荷且让您呼吸较正常更为急促的活动，仅回想您所做过**每次至少10分钟**的那些身体活动，如提重物、苦力、有氧运动或快骑脚踏车等；

**
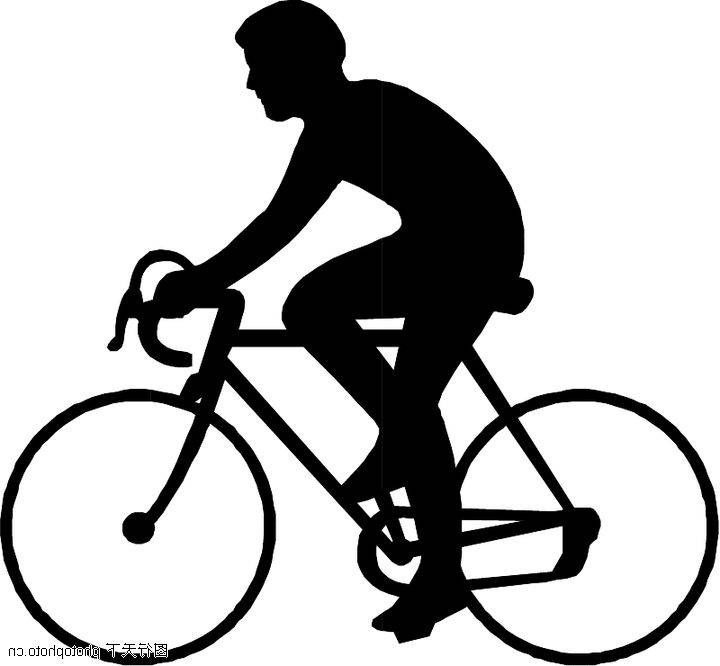
**


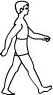
**中等强度活动**是指以适度的身体负荷让您呼吸比正常费力一些的活动，如提轻的物品、正常的速度骑脚踏车或网球双打等，但**不包含走路**；

**走路**包含工作、在家、从某地到某地、娱乐、游戏或休闲时的走路。

**6. 在最近的7天里，您有几天进行以下活动？这7天里，平均每天每项活动的时间分别为？**

|  | 0天 | 1天 | 2天 | 3天 | 4天 | 5天 | 6天 | 7天 | 平均每天活动时间为 |
| --- | --- | --- | --- | --- | --- | --- | --- | --- | --- |
| 剧烈活动 | □ | □ | □ | □ | □ | □ | □ | □ | 小时 分钟 |
| 中等强度活动 | □ | □ | □ | □ | □ | □ | □ | □ | 小时 分钟 |
| 至少走10分钟的路 | □ | □ | □ | □ | □ | □ | □ | □ | 小时 分钟 |

**7. 工作期间（如朝九晚五，8小时），您通常每天静坐的时间约为 小时 分钟。**

**8. 在非工作时间里，您通常每天静坐（主要包括看电视、使用电脑和手机）的时间为？请针对工作日间和周末分别回答**

|  | | 时 间 | 总 计 |
| --- | --- | --- | --- |
| **工**  **作**  **日** | 看电视、电影、阅读 | 约 小时 分钟 | 约 小时 分钟 |
|  | 玩电脑、手机  (浏览网页、聊天、游戏、办公等) | 约 小时 分钟 |  |
|  | 其它（请详写） | 约 小时 分钟 |  |
| **周**  **末** | 看电视、电影、阅读 | 约 小时 分钟 | 约 小时 分钟 |
|  | 玩电脑、手机  (浏览网页、聊天、游戏、办公等) | 约 小时 分钟 |  |
|  | 其它（请详写） | 约 小时 分钟 |  |

**9. 您通常每天睡眠（晚上和午睡）多长时间？ 小时 分钟**

**10. 您在过去的半年内……？ *请在每栏里选择一个答案***

|  | 1） | 2） | 3） |
| --- | --- | --- | --- |
|  | 没有 | 偶尔 | 时常 |
| 说梦话或者梦游吗 | □ | □ | □ |
| 做过噩梦吗？ | □ | □ | □ |
| 入睡困难吗？ | □ | □ | □ |
| 后半夜起来后,再次入睡困难吗？ | □ | □ | □ |
| 入睡容易且能睡整晚并中间不醒来吗？ | □ | □ | □ |
| 经常感觉到疲劳 | □ | □ | □ |
| 行动迟缓，缺乏活力 | □ | □ | □ |

**11. 您与孩子接触的方式主要为**

1□ 谈话 2□ 游戏 3□ 辅导作业 4□ 打电话 5□ 任其自由

6□ 其他

**12. 您与孩子每天接触的时间为？** 请针对工作日和周末分别回答

**周末**

1□ 没有

2□ 大约每天半小时

3□ 大约每天一小时

4□ 大约每天两小时

5□ 大约每天三小时

6□ 大约每天四小时

7□ 大约每天五小时

8□ 大约每天六小时

9□ 大约每天七小时或是更多

**工作日**

1□ 没有

2□ 大约每天半小时

3□ 大约每天一小时

4□ 大约每天两小时

5□ 大约每天三小时

6□ 大约每天四小时

7□ 大约每天五小时

8□ 大约每天六小时

9□ 大约每天七小时或是更多

**父母或监护人问卷回答完毕，谢谢！**

**父母或监护人问卷回答完毕，谢谢！**

**幼儿及家庭情况问卷**

请您和您的配偶或其他监护人一起根据实际情况，回答以下关于**您子女**的问题。如果您是孩子的唯一监护人，则由您个人完成！

**1. 孩子出生时身长    厘米 出生时体重   公斤**

**2. 您觉得孩子的身体健康状况是？**

1□ 非常好 2□ 好 3□ 一般 4□ 差 5□ 很差

**3. 您的孩子经常吃早饭吗?** (不仅仅是一杯牛奶或果汁) 请针对上幼儿园期间和周末分别回答

**上幼儿园期间**

1□ 上幼儿园期间从不吃早饭 2□ 一天 3□ 两天

4□ 三天 5□ 四天 6□ 五天

**周末**

1□ 周末从不吃早饭 2□ 通常在周末吃一次早饭（周六或周日）

3□ 通常在周末都吃早饭（周六和周日）

***4. 您的孩子每周经常吃或喝下面的食物或饮品吗？****请对每种食物选择一个答案*

|  | 从未 | 每周<=1次 | 每周2-4次 | 每周5-6次 | 每天>=1次 |
| --- | --- | --- | --- | --- | --- |
| 1. 水果 | □ | □ | □ | □ | □ |
| 2. 蔬菜 | □ | □ | □ | □ | □ |
| 3. 糖果（糖或巧克力） | □ | □ | □ | □ | □ |
| 4. 可乐或是其它含糖的碳酸软饮料 | □ | □ | □ | □ | □ |
| 5. 牛奶及奶制品 | □ | □ | □ | □ | □ |
| 6. 汉堡包或热狗 | □ | □ | □ | □ | □ |
| 7. 比萨 | □ | □ | □ | □ | □ |
| 8. 方便面 | □ | □ | □ | □ | □ |
| 9.膨化食品（如薯片、妙脆角） | □ | □ | □ | □ | □ |

**5. 您的孩子在读幼儿园之前是否参加过早教（不包括托班）？**

1□ 是 2□ 否

**若选“是”，请问您的孩子是从** **个月（月龄）开始参加早教的，共参加了** **个月的时间 。**

在下面的问题中，您孩子的**体育活动**是指包括各种运动在内的活动，如：走、跑、跳、翻滚，做有肢体运动的游戏等。

**在下面的两个问题中，计算出您的孩子每天在家体育活动的总时间。**

**6-1. 上幼儿园期间（周一至周五），您的孩子平均每天在家体育活动的时间?**

1□ 30分钟以下 2□ 30-60分钟 3□ 1-2小时

4□ 2-3小时 5□ 3-4小时 6□ 4小时及以上

**6-2. 周末时，您的孩子平均每天体育活动的时间?（包括在家及外出）**

1□ 30分钟以下 2□ 30-60分钟 3□ 1-2小时 4□ 2-3小时

5□ 3-4小时 6□ 4-5小时 7□ 5-6小时 8□ 6小时及以上

**7. 上周内您带孩子出去玩的（天数）大约为多久？**

1□ 1天 2□ 2天 3□ 3天 4□ 4天 5□ 5天 6□ 6天 7□ 7天

**8. 您的孩子每周定期参加校外的体育组织活动？**

1□ 0次 2□ 1-3次 3□ 4-6次

**9. 您认为您的孩子现在的体育活动量足够吗？**

1□ 足够 2□ 不够

**10. 您知道体力活动的缺失对孩子的影响吗？**

1□ 不知道 2□ 知道一点 3□ 知道很多

**11. 您认为影响孩子体力活动的障碍在于：*可多选***

1□ 孩子/家长缺乏出去玩的兴趣 2□ 没时间陪孩子玩 3□ 要花钱

4□ 觉得没意思 5□ 没器材或设施 6□ 没有可供孩子玩耍的公共设施

7□ 学校的运动够多了，不需课外额外增加运动量

**12. 您觉得社区内的环境适合儿童进行体力活动的情况是 ？**

1□ 根本没有 2□ 很少 3□一般 4□比较多 5□非常多

**13-1．每周您经常接/送孩子上/下学的交通方式是？**

1□ 步行 2□ 乘坐自行车 3□ 乘地铁/公交车 4□ 乘私家车

**13-2．每天接送孩子在路上花费的时间大约为（单程）？**

1□ 15分钟之内 2□ 15-20分钟 3□ 21-30分钟 4□ ﹥30分钟

**13-3．如果上题中您没有选择“步行”，请回答此题。**

**您没有选择步行送/接孩子的原因是（步行者跳过此题）**

1□ 不安全 2□ 时间紧 3□ 步行很累 4□ 距离远 5□ 孩子体质偏差

6□ 交通状况差 7□ 人行道路面条件差 8□ 空气质量差

**14. 您的孩子在家（幼儿园上学时间之外）每天有几个小时看电视电影、玩电脑电子游戏或写作业？**请针对上学日和周末分别回答

|  | | 时 间 | 总 计 |
| --- | --- | --- | --- |
| 上  学  日 | 看电视、电影、阅读 | 小时 分钟 | 小时 分钟 |
|  | 玩电脑、手机  (浏览网页、聊天、游戏、办公等) | 小时 分钟 |  |
|  | 写作业 | 小时 分钟 |  |
|  | 其他（请详写） | 小时 分钟 |  |
| 周  末 | 看电视、电影、阅读 | 小时 分钟 | 小时 分钟 |
|  | 玩电脑、手机  (浏览网页、聊天、游戏、办公等) | 小时 分钟 |  |
|  | 写作业 | 小时 分钟 |  |
|  | 其他（请详写） | 小时 分钟 |  |

请回答以下关于您孩子睡眠的问题

**15. 您的孩子通常每天睡眠（晚上和午睡）多长时间？ 小时 分钟**

**16. 在过去的半年内，您的子女…… *请在每栏里选择一个答案***

|  | 1） | 2） | 3） |
| --- | --- | --- | --- |
|  | 没有 | 偶尔 | 时常 |
| 不愿独自睡觉吗 | □ | □ | □ |
| 说梦话或者梦游吗 | □ | □ | □ |
| 做过噩梦吗？ | □ | □ | □ |
| 入睡困难吗？ | □ | □ | □ |
| 后半夜起来后,再次入睡困难吗？ | □ | □ | □ |
| 入睡容易且能睡整晚并中间不醒来吗？ | □ | □ | □ |
| 比大多数孩子都睡得少吗 | □ | □ | □ |
| 经常感觉到疲劳 | □ | □ | □ |
| 行动迟缓，缺乏活力 | □ | □ | □ |

**17. 孩子亲生父母目前的婚姻情况？**

1□ 保持正常婚姻 2□ 单亲尚未再婚且孩子为父亲抚养

3□ 单亲尚未再婚且孩子为母亲抚养 4□ 单亲已再婚且孩子为父亲抚养

5□ 单亲已再婚且孩子为母亲抚养 6□ 其他：

18、19题是关于孩子**看护人**情况的问题。

**看护人界定标准**：某段时间内参与看护孩子的总时间为1个月以上。

**18. 孩子成长过程中的看护情况** *可多选*

| 年龄  阶段 | 父母  独立看护 | 祖辈  独立看护 | 父母、祖辈  共同看护 | 父母、保姆  共同看护 | 祖辈、保姆  共同看护 | 父母、祖辈、保姆  共同看护 | 其他： |
| --- | --- | --- | --- | --- | --- | --- | --- |
| 0-0.5岁 | □ | □ | □ | □ | □ | □ | □ |
| 0.5-1岁 | □ | □ | □ | □ | □ | □ | □ |
| 1-1.5岁 | □ | □ | □ | □ | □ | □ | □ |
| 1.5-2岁 | □ | □ | □ | □ | □ | □ | □ |
| 2-2.5岁 | □ | □ | □ | □ | □ | □ | □ |
| 2.5-3岁 | □ | □ | □ | □ | □ | □ | □ |
| 3-3.5岁 | □ | □ | □ | □ | □ | □ | □ |
| 3.5-4岁 | □ | □ | □ | □ | □ | □ | □ |
| 4岁以后 | □ | □ | □ | □ | □ | □ | □ |

**19. 孩子看护人的基本情况** *请认真阅读以下说明后再填写*

说明：

1. 若下列表中“看护人”一列中的某人并没有担任过孩子出生以来的看护人，该人所在一行就不用填写；
2. 若某看护人已经去世，仍需填写他/她的各项信息。现在年龄是指若生活到现在，他/她所达到的年龄；
3. 学历：1.小学及以下 2.初中 3.高中或中专 4.大专 5.本科 6.硕士研究生 7.博士研究生。
4. 体育活动是指各类体育项目运动与有目的的健身活动。
5. “对体育活动的喜爱程度”是指喜欢观赏体育比赛或体育节目，或期望参加体育活动的程度等。

| 看护人 | 现在年龄 | 学历 | 对体育活动的参与程度 | | | | |  | | 对体育活动的喜爱程度 | | | | | |
| --- | --- | --- | --- | --- | --- | --- | --- | --- | --- | --- | --- | --- | --- | --- | --- |
|  |  |  | 每周  6次及以上 | 每周3-5次 | 每周1-2次 | 每周1次以下 | 几乎不参加 | |  | | 非常  喜欢 | 比较  喜欢 | 一般 | 比较  不喜欢 | 非常  不喜欢 |
| 父亲 |  |  | □ | □ | □ | □ | □ | |  | | □ | □ | □ | □ | □ |
| 母亲 |  |  | □ | □ | □ | □ | □ | |  | | □ | □ | □ | □ | □ |
| 爷爷 |  |  | □ | □ | □ | □ | □ | |  | | □ | □ | □ | □ | □ |
| 奶奶 |  |  | □ | □ | □ | □ | □ | |  | | □ | □ | □ | □ | □ |
| 外公 |  |  | □ | □ | □ | □ | □ | |  | | □ | □ | □ | □ | □ |
| 外婆 |  |  | □ | □ | □ | □ | □ | |  | | □ | □ | □ | □ | □ |
| 保姆 |  |  | □ | □ | □ | □ | □ | |  | | □ | □ | □ | □ | □ |

请回答以下关于居住小区及周边环境的问题

**20. 您居住的小区及周边环境（请在最符合现状的空格内“√”）**

| 居住小区及周边环境 | 完全符合 | 基本符合 | 说不清楚 | 不太符合 | 完全不符合 |
| --- | --- | --- | --- | --- | --- |
| 1.道路拥挤 |  |  |  |  |  |
| 2.道路路面情况不佳 |  |  |  |  |  |
| 3.缺少活动场地 |  |  |  |  |  |
| 4.治安不好 |  |  |  |  |  |
| 5.周边快餐店很多 |  |  |  |  |  |
| 6.绿化不好 |  |  |  |  |  |
| 7.卫生情况差 |  |  |  |  |  |
| 8.人员素质差 |  |  |  |  |  |
| 9.养宠物的很多 |  |  |  |  |  |
| 10.小区人行道和机动车混合 |  |  |  |  |  |
| 11.每天坐电梯上下楼 |  |  |  |  |  |

**21. 您去的场所（如下）可能要花费的时间（请在最符合您目前情况的选项上打“√”）**

| 居住地生活服务设施 | 无此场所 | ＜10  分钟 | 11-20  分钟 | 21-30  分钟 | ＞30  分钟 | 不知道 |
| --- | --- | --- | --- | --- | --- | --- |
| 广场 |  |  |  |  |  |  |
| 公园／绿地 |  |  |  |  |  |  |
| 大型超市／大型商场 |  |  |  |  |  |  |
| 幼儿园 |  |  |  |  |  |  |
| 步行街／商业街 |  |  |  |  |  |  |
| 儿童娱乐场所 |  |  |  |  |  |  |
| 体育场馆 |  |  |  |  |  |  |
| 步行／郊游／自行车路径 |  |  |  |  |  |  |
| 最近的亲戚／朋友家 |  |  |  |  |  |  |

**问卷到此结束，再次感谢您及您家庭的参与！祝您愉快！**
